# Validation of oral-nasal specimen collection for influenza and respiratory syncytial virus detection

**DOI:** 10.1017/ash.2025.66

**Published:** 2025-04-23

**Authors:** Matthew Young, Yuri Suico, Omid Kyle Vojdani, Janine McCready, Kevin Katz, Scarlett Pourmatin, Manija Rahimi, Christie Vermeiren, Jeff Powis, Christopher Kandel

**Affiliations:** 1 Department of Medicine, Michael Garron Hospital, University of Toronto, Toronto, ON, Canada; 2 Department of Laboratory Medicine, University of Toronto, Toronto, ON, Canada; 3 Shared Hospital Laboratory, Toronto, ON, Canada; 4 North York General Hospital, Toronto, ON, Canada; 5 Faculty of Medicine, University of Toronto, Toronto, ON, Canada

## Abstract

**Objective:**

Respiratory virus testing is routinely performed and ways to obtain specimens aside from a nasopharyngeal swab are needed for pandemic preparedness. The main objective is to validate a self-collected oral-nasal swab for the detection of Influenza and respiratory syncytial virus (RSV).

**Design:**

Diagnostic test validation of a self-collected oral nasal swab as compared to a provider-collected nasopharyngeal swab.

**Setting:**

Emergency Department at Michael Garron Hospital.

**Participants:**

Consecutive individuals who presented to the Emergency Department with a suspected viral upper respiratory tract infection were included if they self-collected an oral-nasal swab. Individuals testing positive for Influenza or RSV along with randomly selected participants who tested negative were eligible for inclusion.

**Interventions:**

All participants had the paired oral-nasal swab tested using a multiplex respiratory virus polymerase chain reaction for the three respiratory pathogens and compared to the nasopharyngeal swab.

**Results:**

48 individuals tested positive for Influenza, severe acute respiratory coronavirus virus 2 (SARS-CoV-2) or RSV along with 80 who tested negative. 110 were symptomatic with the median time from symptom onset to testing of 1 day (interquartile range 2–5 days). Using the clinical nasopharyngeal swab as the reference standard, the sensitivity was 0.75 (95% CI, 0.43–0.95) and specificity was 0.99 (95% CI, 0.93–1.00) for RSV, sensitivity is 0.67 (95% CI, 0.49–0.81) and specificity is 0.96 (95% CI, 0.89–0.99) for Influenza.

**Conclusions:**

Multiplex testing with a self-collected oral-nasal swab for Influenza and RSV is not an acceptable substitute for a healthcare provider collected nasopharyngeal swab primarily due to suboptimal Influenza test characteristics.

## Introduction

Viral testing is routinely performed to determine the cause of symptoms compatible with an upper respiratory tract infection. As numerous respiratory viruses circulate simultaneously and lead to similar clinical manifestations, identifying the causative pathogen is needed for ensuring appropriate treatments are provided in a timely fashion and to inform transmission prevention measures. As the armamentarium of anti-viral therapies expands, there has been a shift towards multiplex panels whereby multiple viral targets are tested from a single clinical specimen. A barrier to viral testing has been the need for specimen collection from the nasopharynx, which is not only uncomfortable but cannot be self-collected.^1^

During the COVID-19 pandemic, alternative testing strategies were evaluated for severe acute respiratory coronavirus virus 2 (SARS-CoV-2) testing.^2,3^ The oral-nasal collection technique – swabbing both anterior nares along with the tongue and buccal mucosa with a flocked swab – was evaluated for SARS-CoV-2, which found that a self-collected oral-nasal swab had comparable accuracy to a healthcare provider collected nasopharyngeal swab.^3^ Combined oral-nasal swabbing was further extended to rapid antigen testing for SARS-CoV-2 and found to be similarly robust.^4^ Self-collected mid-turbinate swabs have previously been found to be accurate when testing other respiratory pathogens. including Influenza and respiratory syncytial virus (RSV).^5,6^ Mid-turbinate swabs can be challenging for children or individuals with cognitive impairment to collect properly and more straightforward collection strategies are needed. Saliva has been evaluated for numerous viral pathogens, but is limited by varying proprietary collection containers, lack of standard testing protocols and the inability of some individuals to produce sufficient saliva when ill.^7^ We sought to determine the performance characteristics of a self-collected oral-nasal swab for Influenza or RSV as part of a multiplex viral testing panel as compared to a healthcare provider collected nasopharyngeal swab.

## Methods

### Study population

The study population was consecutive adults (age > 18) who presented to the ambulatory zone of the Emergency Department at Michael Garron Hospital in Toronto, Ontario, Canada who had a nasopharyngeal swab for respiratory pathogens collected as part of routine care and consented to the self-collection of a paired oral-nasal swab. The study period ran from January 18, 2023 until March 31, 2023 and October 28, 2023 until March 6, 2024 when multiplex testing for severe acute respiratory coronavirus virus 2 (SARS-CoV-2), Influenza A, Influenza B and RSV was available. For the self-collected oral-nasal swab, individuals self-swabbed the anterior aspect of both nares, the buccal mucosa, and the tongue using a disposable flocked swab.^3^ All oral-nasal swabs were stored at 4^o^C until testing. This study was approved by the Research Ethics Board at Michael Garron Hospital (Reference Number: NR-349).

### Study design

Once the clinical nasopharyngeal swab results were reported positive for a study participant for one of Influenza A, Influenza B or RSV, the paired oral-nasal swab and, up to two randomly selected negative oral-nasal swabs from individuals with a negative clinical specimen from the same day or day prior to the positive specimen were subjected to multiplex testing. Clinical information (age, self-reported sex and presence of viral symptoms, cough or fever) for all participants was abstracted by chart review.

### Influenza and RSV detection

All specimens were processed at Shared Hospital Laboratory (Toronto, ON). All swab samples were placed into Copan Universal Transport Media. A 160-µl aliquot was extracted in the Hamilton Star automated extraction instrument, using the Maxwell® HT Viral TNA Kit (Promega). Detection of viral targets was performed using a laboratory-developed real-time reverse-transcription polymerase chain reaction (RT-PCR) assay for the detection of Influenza A, Influenza B, and RSV in addition to SARS-CoV-2, Adenovirus, Parainfluenza Virus 1–4, Coronavirus OC43/229E/NL63/HKU-1, Rhino/Enterovirus, Human Metapneumovirus and internal control (RNAseP) using the Luna Universal Probe One-Step RT q-PCR kit (New England Biolabs) on the CFX96 Touch Real-Time PCR detection system (BioRad). Positive specimens were defined as those with a cycle threshold (Ct) for the viral target below 37. Validation specimens were processed using the same protocols and tested concurrently.

### Statistical analysis

The cohort characteristics were presented with means or medians for continuous variables and proportions for categorical variables. Performance characteristics (sensitivity and specificity) were calculated for each virus separately using the healthcare provider-collected nasopharyngeal swab as the reference standard. The kappa coefficient was used to estimate the agreement between the nasopharyngeal and self-collected oral-nasal swabs. Cycle threshold value results between the clinical and validation specimens for each virus were displayed graphically. All analyses were performed using R version 4.2 (R Foundation for Statistical Computing, Vienna, Austria).

## Results

Over the study period, there were 128 individuals who provided a paired oral-nasal specimen that was processed for validation testing; 29 with Influenza A, 7 with Influenza B, 12 with RSV and 80 who were negative (10 detected other respiratory pathogens). The median age was 49 (interquartile range 32–69) and 46% (59/128) were female. Of the 94% (120/128) with clinical information available, 92% (110/120) were symptomatic at the time of testing. Of the 110 with symptoms, the median time from symptom onset to testing was 1 day (interquartile range 2–5 days), 51% (56/110) had fever and 76% (84/110) reported a cough.

**Figure 1.**
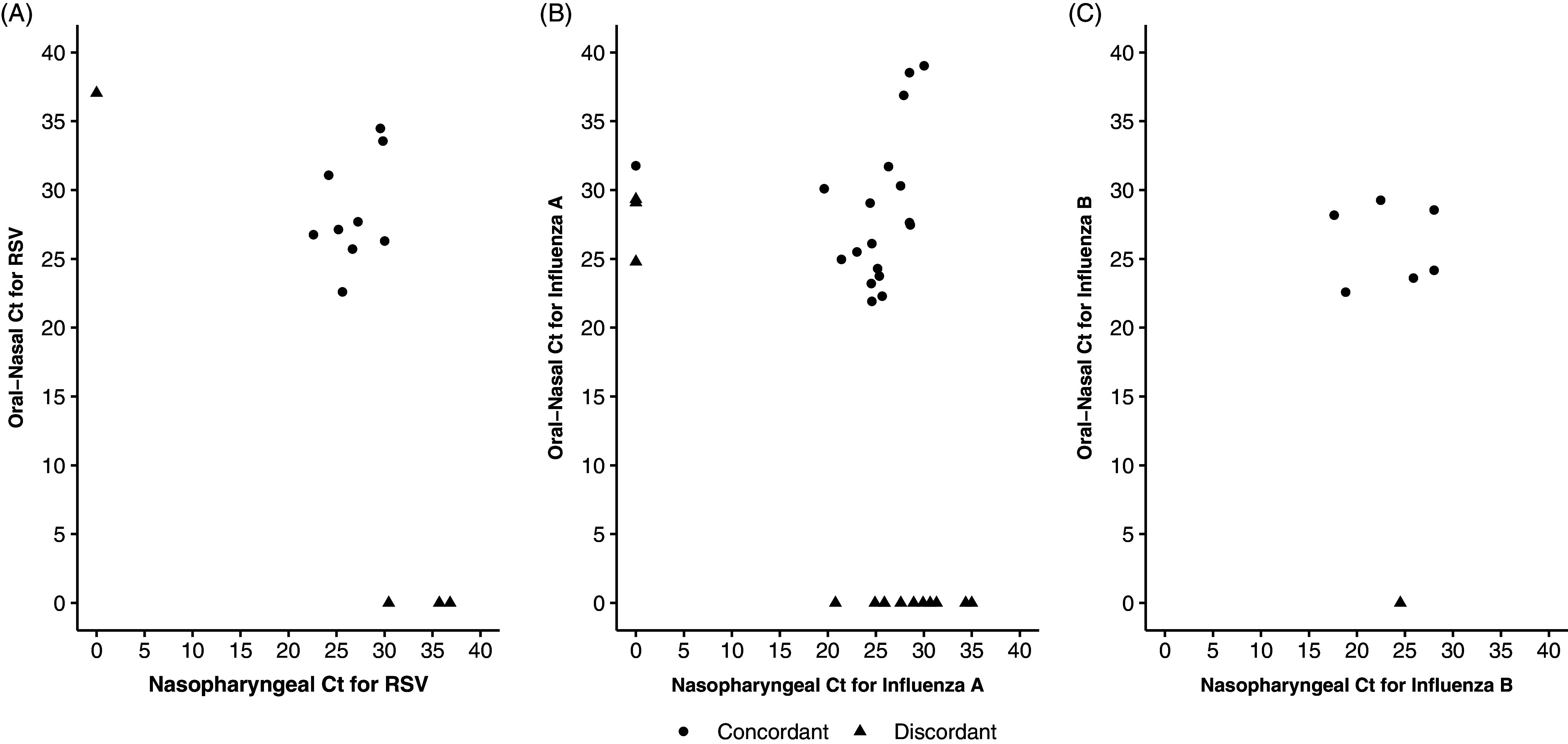
Cycle threshold values for the healthcare provider collected clinical nasopharyngeal swab and the paired self-collected combined oral-nasal swab for respiratory syncytial virus (A), influenza A (B) and influenza B (C). One nasopharyngeal swab for Influenza A did not have a cycle threshold available.

With the nasopharyngeal swab as the reference standard, the sensitivity was (0.75 [95% CI, 0.43–0.95]) and specificity is (0.99 [95% CI, 0.93–1.00]) for RSV (Table 1). For combined Influenza A and Influenza B the sensitivity is (0.67 [95% CI, 0.49–0.81]) and specificity is (0.96 [95% CI, 0.89–0.99]). The percent agreement between the nasopharyngeal swab and the oral-nasal swab for RSV was (0.96 kappa coefficient 0.79 [95% CI, 0.56–0.92]) and for Influenza was (0.87 kappa coefficient 0.68 [95% CI 0.52-0.80]). There was one discordant nasopharyngeal swab for RSV and three for Influenza A. For paired RSV specimens, the discordant oral-nasal swabs were above a cycle threshold of 30 whereas for Influenza the discordant swabs were observed at lower cycle thresholds (Figure 1).

**Table 1.** Performance characteristics of respiratory syncytial virus and influenza detection in paired nasopharyngeal swab and oral-nasal swab specimens from individuals presenting to the emergency department

		Nasopharyngeal swab	
Virus	Oral-nasal swab	Positive	Negative
Respiratory syncytial virus	Positive	9	1
	Negative	3	79
Influenza	Positive	24	3
	Negative	12	77

## Discussion

In ambulatory zones of an Emergency Department a self-collected oral-nasal swab for respiratory viruses is feasible, required minimal instruction and avoided the presence of a healthcare professional. When compared to a healthcare provider-collected nasopharyngeal swab the combined oral-nasal swab is not a comparable alternative for multiplex testing of RSV and Influenza primarily due to suboptimal Influenza test characteristics.

Ongoing RSV testing is needed to determine the effectiveness of immunization and infant monoclonal antibody programs and to avoid unnecessary antibiotics. In a prospective cohort of adults who were pregnant, testing for RSV using a mid-turbinate swab was compared to a nasopharyngeal swab and found a sensitivity of (0.94 [95% CI, 0.71–1.00]) and specificity (0.99 [95% CI, 0.97–1.00]) in a cohort with 16 positive specimens.^5^ Similarly, when comparing various specimen collection methods among adults admitted to hospital with suspected pneumonia, the sensitivity for RSV was higher for saliva and sputum specimens (0.72 and 0.70, respectively) than a nasopharyngeal swab (0.51).^8^ This may explain the preserved performance characteristics and the false negative nasopharyngeal swab in this study as RSV shedding seems to be detectable in both the nasopharynx and oropharynx.

Non nasopharyngeal specimen collection techniques have been evaluated for the Influenza virus. Paired nasopharyngeal and mid-turbinate swabs were evaluated in adult outpatients with a suspected viral upper respiratory tract infection. One study found that a mid-turbinate swab had a sensitivity of (1.00 [95% CI, 0.40–1.00]) for Influenza, albeit with only 3 positive specimens and with the reference standard as a positive specimen from any collection type.^6^ In a cohort of adults assessed in an Emergency Department, saliva was compared to nasopharyngeal specimens with 19 positive Influenza specimens available for evaluation. The sensitivity of saliva was found to be (0.90 [95% CI, 0.73–1.00]).^9^ In contrast, when comparing self-collected nasal swabs to provider-collected nasopharyngeal swabs in older adults the sensitivity was (0.78 [95% CI, 0.40–0.97]).^10^ In this study cohort, the combination of swabbing the anterior nares with that of the oropharynx did not seem to improve detection of Influenza.

For both Influenza and RSV the resulting cycle thresholds – a surrogate for viral load – tended to be higher in the self-collected oral-nasal specimen as compared to the paired nasopharyngeal swab, which is similar to that observed with SARS-CoV-2 .^3^ This observation was similarly observed when evaluating saliva for Influenza testing, which found that median cycle threshold values were lower in the paired nasopharyngeal swab.^9^ Given that viral loads decline over time, it is likely that the performance characteristics of the oral-nasal swab will be more unfavorable as the time from symptom onset increases.

There are limitations of this study. First, the sample size is relatively small leading to less precise estimates of the performance characteristics of the oral-nasal swab. However, the number of positive specimens were more numerous than most published diagnostic validation studies. Second, the study population consisted of individuals with a suspected viral illness who were experiencing relatively mild symptoms that began shortly before specimen collection so the performance characteristics of the oral nasal swab in the setting of a prolonged duration of symptoms are unknown. Third, the study population of this cohort comprised solely of adults and the results may not apply to children where alternative collection techniques to a nasopharyngeal are preferred.

In conclusion, this study was the first to report the performance characteristics of the oral-nasal swab for multiplex viral testing. Self-collected oral-nasal swabs are not an ideal option for RSV and Influenza when testing adults presenting to the Emergency Department with a suspected upper respiratory viral infection. Nasopharyngeal swabs should remain the standard of care for the time being while additional testing collection techniques are evaluated.
